# The Design of a Circularly Polarized Antenna Array with Flat-Top Beam for an Electronic Toll Collection System

**DOI:** 10.3390/s23239388

**Published:** 2023-11-24

**Authors:** Tianfan Xu, Mengchi Xu, Xiao Cai

**Affiliations:** Research Center of Applied Electromagnetics, Nanjing University of Information Science and Technology, Nanjing 210044, China; xu18121413148@126.com (T.X.); xumengchi01@163.com (M.X.)

**Keywords:** electronic toll collection system, flat-top beam, method of the maximum power transmission efficiency

## Abstract

Electronic toll collection (ETC), known as a non-stop toll collection system which can automatically realize payment by setting the identification antenna at the entrance, is always suffering from information exchange interruption caused by beam switching. A circularly polarized sector beam antenna array operating at 5.8 GHz with flat-top coverage is proposed, based on the weighted constrained method of the maximum power transmission efficiency (WCMMPTE). By setting the test receiving antennas at the specific angles of the ETC antenna array to be designed, constraints on the received power are introduced to control the radiation pattern and obtain the optimized distribution of excitations for antenna elements. A 1-to-16 feeding network, based on the microstrip transmission line theory is designed to feed a 4 × 4 antenna array. Simulation results show that the half-power beamwidth covers an angular range of −30° to 30° while the axial ratio is below 3dB, which meets the ETC requirements. Furthermore, the gain fluctuation among the needed range of −30° to 30° is lower than 0.7 dB, which is suitable for the ETC system to achieve a stable signal strength and uninterrupted communication.

## 1. Introduction

Antenna arrays with flat-top beam, which means stable gain with extremely low fluctuation in the required region, are widely applied in base stations [1,2,3], radio frequency identification [4,5,6], electronic toll collection [7,8,9,10,11,12], and other wireless communication systems [13,14,15,16,17,18,19,20,21,22].

Based on beam-forming techniques or optimization algorithms, the antenna array can generate desired radiation patterns with an optimized distribution of amplitudes and phases for the antenna elements. Commonly used beam-forming methods include the Woodward–Lawson method [23], the Dolph–Chebyshev method [24,25], and the Fourier transform method [26,27,28]. Based on exploiting the nature of the continuous aperture distribution, by expressing it as a Fourier series of moderately high orders, an improved pattern synthesis iterative method in planar arrays was put forward in [28]. Li et al. presented a flat-top beam-shaping method which simplifies the optimization process by combining the Taylor and Woodward–Lawson methods in [29]. A digital technique to accomplish the fixed amplitude and phase distribution to create the beam was proposed in [30], which provided the possibility of dynamic high-speed changes for the beam shape.

Except for the classical methods, optimization algorithms also play an important role in the design of beam-forming antenna arrays. The genetic algorithm [31,32,33], particle swarm optimization [34,35], simulated annealing [36] and other algorithms [37,38,39] are used to adjust radiation patterns to the desired shapes. Mansutti, Carlo and Hannan offered a transmit-array with beam scanning capabilities by using both particle swarm optimization and a genetic algorithm to optimize the design of an antenna. It should be noted here the iteration time will increase when the number of antenna elements increases. the A zero placement algorithm for the synthesis of a flat-top beam pattern with low sidelobe level (SLL) was discussed in [37], which is able to synthesize a flat-top and narrow main beam with low SLL directly in the Z domain, by breaking the array factor into two separate parts. Lin et al. proposed a new differential evolution algorithm for unequally spaced antenna arrays to accomplish a flat-top beam [38]. The case of non-uniformly distributed antennas is also proposed in [40].

However, the above-mentioned traditional methods rely on the array factor, which is unavailable in antenna array designs with irregular array configurations, small inter-element spacing and complex environments. Thus, new methods for antenna array designs should be further explored.

In this work, an optimization theory for the design of beam-shaping antenna arrays, which is called the weighted constrained method of the maximum power transmission efficiency (WCMMPTE), is proposed. While the main function ensures the optimal antenna array performance, the constraints can be various, depending on the practical requirements. By introducing equality constraints, the flat-top beam is shaped with an extremely low gain fluctuation. A 16-element antenna array is designed to validate the beam-forming effect of the proposed WCMMPE. Simulation results show that a flat-top sector beam is generated by feeding the antenna elements with the optimized distribution of excitations, covering the whole required area. Compared with the existing beam-switching mechanisms, the proposed design based on WCMMPTE provides a more stable signal during uninterrupted communication, which is suitable for the electronic toll collection (ETC) system.

## 2. Optimization Theory

Consider a general multiple-input multiple-output (MIMO) system, which consists of an *m*-element transmitting array to be designed and *n* test receiving antennas. The normalized incident and reflected power waves for the transmitting and receiving antennas are denoted by

(1) $$\begin{array}{l} {}[a_{t}]={\left[a_{1},a_{2},\ldots ,a_{m}\right]}^{T},\\ {}[a_{r}]={\left[a_{m+1},a_{m+2},\ldots ,a_{m+n}\right]}^{T},\\ {}[b_{t}]={\left[b_{1},b_{2},\ldots ,b_{m}\right]}^{T},\\ {}[b_{r}]={\left[b_{m+1},b_{m+2},\ldots ,b_{m+n}\right]}^{T}, \end{array}$$

Here, the superscript ‘*T*’ indicates the matrix transposition, while the subscript ‘*t*’ and ‘*r*’, respectively, stand for transmitting and receiving. Then, the transmitting array and the test receiving antennas can be viewed as a multiple-input multiple-output system and characterized by the scattering parameters, as follows:

(2) $$\left[\begin{array}{l} \left[b_{t}\right]\\ \left[b_{r}\right] \end{array}\right]=\left[\begin{array}{l} \left[S_{tt}]\;[S_{t}{}_{r}\right]\\ \left[S_{rt}]\;[S_{tt}\right] \end{array}\right]\;\left[\begin{array}{l} \left[a_{t}\right]\\ \left[a_{r}\right] \end{array}\right],$$

Hence, the power transmission efficiency $\eta_{array}$ between the transmitting array and the receiving antennas is defined as the ratio of the power delivered to the loads of the receiving array to the input power to the transmitting array, expressed as:

(3) $$\eta_{array}=\frac{\frac{1}{2}({\left|\left[b_{r}\right]\right|}^{2}-{\left|\left[a_{r}\right]\right|}^{2})}{\frac{1}{2}({\left|\left[a_{t}\right]\right|}^{2}-{\left|\left[b_{t}\right]\right|}^{2})}.$$

If the test receiving antennas are well-matched, we may have $[a_{r}]=0$, and (3) yields

(4) $$\eta_{array}=\frac{{\left[a_{t}\right]}^{H}[A][a_{t}]}{{\left[a_{t}\right]}^{H}[a_{t}]},$$

where $A={\left[S_{rt}\right]}^{H}[S_{rt}]$ and the subscript ‘*H*’ represents the Hermitian operation (conjugate transpose). 

For multiple test receiving antennas, the received power of each test antenna can be expressed by ${\left[b_{r}\right]}^{2}$. In order to shape the radiation pattern, the test receiving antennas are all placed on the sphere of the transmitting antenna array, with a radius *r*. It should be noted that the test receiving antennas are set in the far-field region, so that *r* must meet the condition expressed as

(5) $$r\geq \frac{2D^{2}}{\lambda },$$

where *D* is the aperture of the transmitting antenna array and λ is the wavelength in free space. If the test receiving antenna is omni-directional, constraints on the received power of each test antenna are equal to the gain of the transmitting antenna array at the position where the test antenna is placed. In order to achieve a flat-top sector beam, several test antennas should be uniformly distributed among the angular range to be shaped. Then, the optimal design problem can be formulated as a quadratically constrained quadratic programing (QCQP), expressed as

(6) $$\begin{array}{l} \max\;\frac{{\left[a_{t}\right]}^{H}[A][a_{t}]}{{\left[a_{t}\right]}^{H}[a_{t}]}\\ s.t.\;\;\;\;{\left|b_{i}\right|}^{2}=c,\;i=1,2,\ldots ,n. \end{array}$$

Here, *c* is a constant, leading to the same received power for all the test antennas. By applying the Lagrange multiplier method, the solution to (6) is in an analytical form

(7) $${\left[a_{t}\right]}^{*}=A^{-1}S^{H}{\left(SA^{-1}S^{H}\right)}^{-1}y,$$

where y represents an *n*-dimensional vector, where each element value is 1. Thus, ${\left[a_{t}\right]}^{*}$ gives the optimal distribution of excitations, which can then be realized by a feeding network or by RF circuits. To sum up, the overall optimal design procedure for the flat-top sector beam antenna array with desired angular coverage is summarized into four steps:

(1) Determine the configuration of the antenna array to be designed. The antenna element, array size, and the inter-element spacing can be determined according to actual requirements or empirical rules. It should be noted that the inter-element spacing is not limited to only half-wavelengths. Hence, the smaller or larger inter-element spacing is applicable for the antenna array design, based on the proposed method.
(2) Set the test receiving antennas at proper positions. In general, the test receiving antennas should evenly cover the area that needs to be shaped. Based on the angular coverage of the area to be shaped, the number of test receiving antennas may vary from case to case, and should be carefully decided. Thus, the shaping area is divided into many small areas. If the number of test receiving antennas is too small, a depression may occur in the curve of gain. Therefore, dividing the target area into two or four parts and placing the receiving antennas at the junctions and edges may be a suitable choice, according to the simulation experiences.
(3) Calculate the scattering parameters. While the arrangement of the antenna array to be designed and test antennas is determined, the multiple-input multiple-output system is formed. A full-wave simulation can be conducted using commercial simulation software, such as HFSS, CST, and FEKO. As a result, the simulated scattering matrix is obtained and used for further optimization.
(4) Obtain the optimal excitations for the antenna array to be designed. Once the scattering matrix is achieved, the optimal amplitudes and phases for the transmitting antenna elements can be obtained using (7). The optimal distribution of excitations can then be accomplished via a feeding network or RF circuits depending on the actual requirements.

## 3. Optimal Antenna Array Design

### 3.1. Antenna Element Design

**Figure 1:**
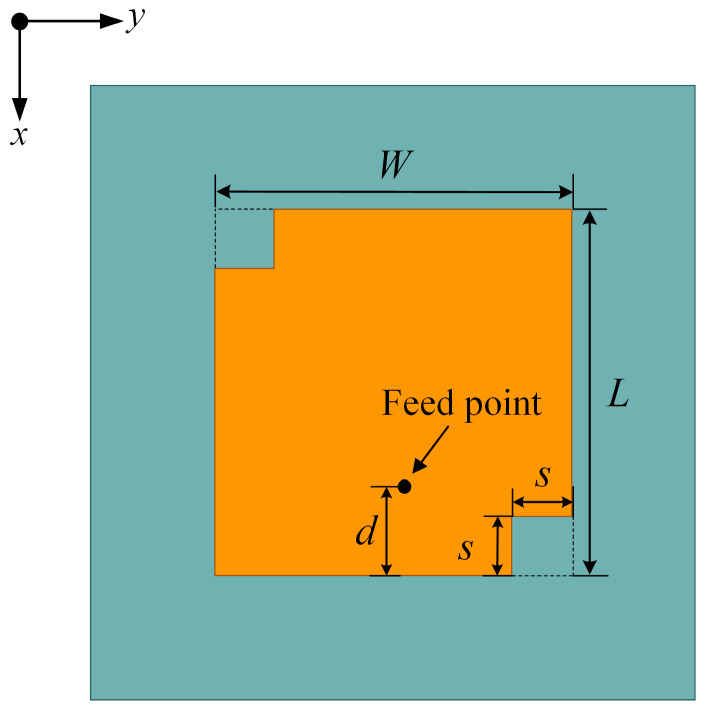
Scheme of the proposed microstrip patch antenna.

A microstrip patch antenna is selected as the array element for its simple structure, light weight, low profile, small size, and low cost, which is suitable for an ETC system. As is shown in Figure 1, a circularly polarized center-fed microstrip antenna element operating at 5.8 GHz is designed on an FR4 dielectric substrate, with a thickness of 2 mm, relative dielectric constant of 4.4, and loss tangent of 0.02. The length and width of the rectangular patch are *W* and *L*. In order to achieve better circular polarization performance, two squares with a side length *s* are, respectively, cut at the upper left corner and lower right corner to generate orthogonal current paths. The distance between the feed point along the x-axis and the lower edge of the patch is *d*. The optimized values for the abovementioned parameters are listed in Table 1.

**Figure 2:**
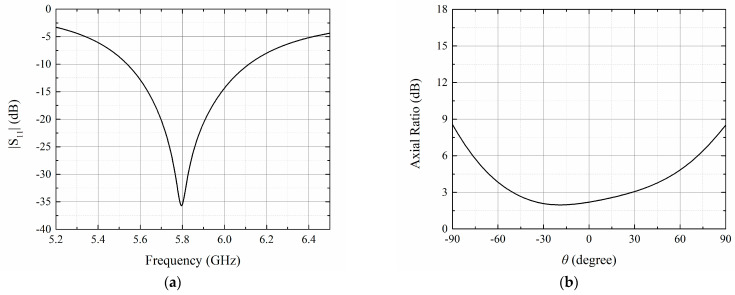
Simulation results of the microstrip patch antenna. (**a**) Reflection coefficient; (**b**) Axial ratio at *yz*-plane.

As illustrated in Figure 2, the absolute bandwidth of the proposed antenna is 579 MHz (from 5.538 GHz to 6.117 GHz). The reflection coefficient at 5.8 GHz is below −35 dB. The simulated axial ratio (AR) at the boresight direction is 2.1 dB, and the good circular polarization characteristic is maintained within the angular coverage from −51° to 29°, which is suitable to form an antenna array.

### 3.2. Antenna Array Design and Excitation Optimization

**Figure 3:**
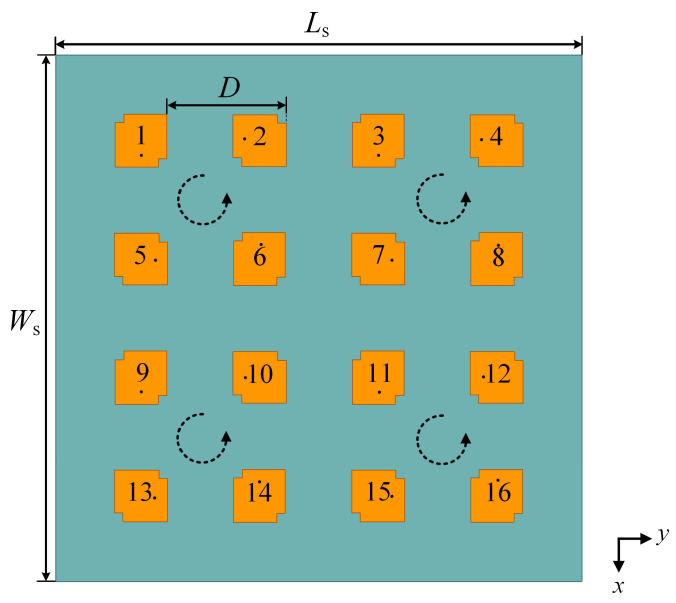
Scheme of the 16-element antenna array.

To pursue higher gain and shape the desired flat-top beam, a 16-element planar antenna array is designed and optimized. As is shown in Figure 3, 16 antenna elements form a 4 × 4 square array with a uniform inter-element spacing *D* of $\lambda_{0}/2$ (where $\lambda_{0}$ is the wavelength in free space). The antenna array has a length *L*_s_ of 114.7 mm and a width *W*_s_ of 115 mm. Noted here, the square array is decomposed into four sequentially rotated sub-arrays to achieve a lower AR and a broader AR bandwidth.

**Figure 4:**
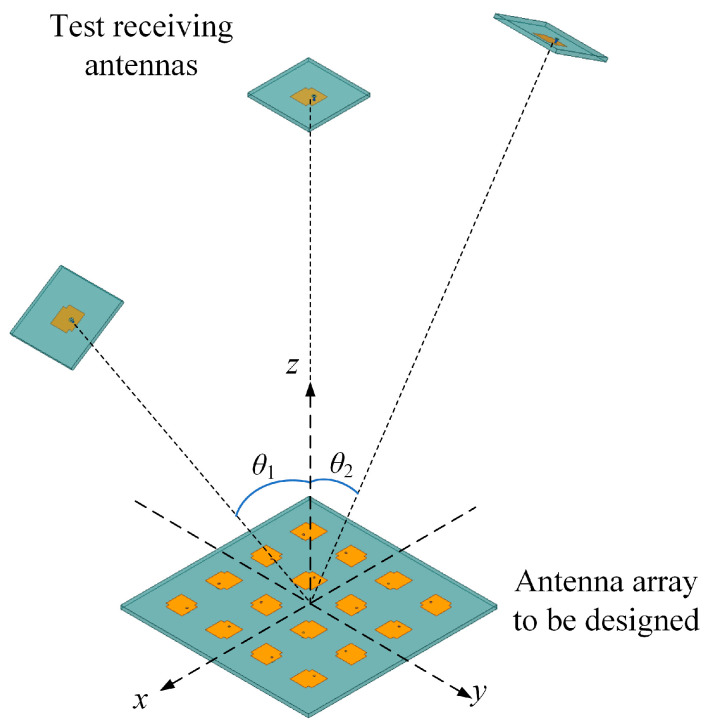
Scheme of the MIMO system with 16 transmitting antennas and 3 receiving antennas.

While the antenna array configuration is determined, three test receiving antennas are arranged in the far-field, which is calculated to be over 562.2 mm according to (5). It can be seen from Figure 4 that all three test antennas are in the *xz*-plane, while the angles of the intervals between bilateral antennas and middle antenna are $\theta_{1}$ and $\theta_{2}$, respectively. Hence, the 16-to-3 MIMO system is formed, and the scattering matrix can be obtained by conducting a full-wave simulation in High Frequency Simulator Structure (HFSS).

**Figure 5:**
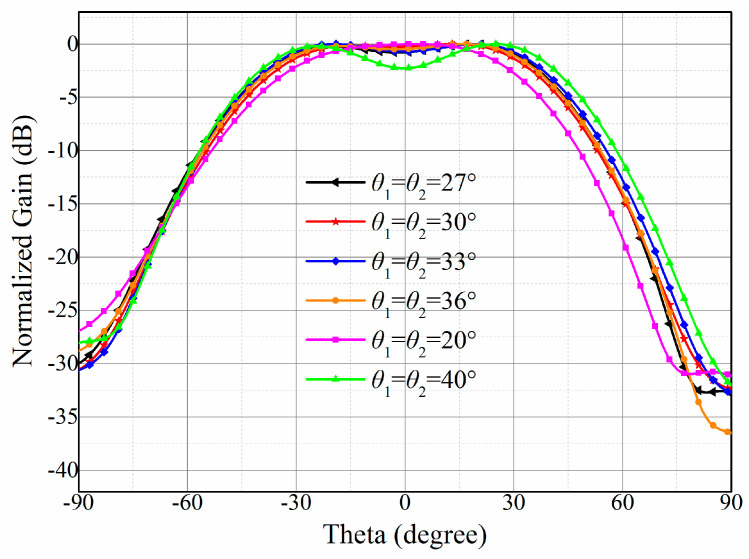
Radiation patterns of the *xz*-plane with the changing positions of test receiving antennas.

Once the scattering parameters are achieved, the optimal distribution of excitations to accomplish the flat-top sector beam is given by (7). Due to the desired angular coverage of −30° to 30°, here, we choose the same value for $\theta_{1}$ and $\theta_{2}$ to ensure the symmetry of the shaped beam. As previously mentioned, the included angle among the test receiving antennas should not be too large or too small. It can be seen in Figure 5 that if the included angle is too small (such as 20°), the coverage of flat-top beams will be narrower than the requirements. If the included angle is too large (such as 40°), there will be a noticeable depression in the middle of the beam, leading to the large gain fluctuation over the desired coverage. In general, the selection of theta determines the beamwidth of the flat-top beam.

Since the values for $\theta_{1}$ and $\theta_{2}$ have significant impacts on the results, we use gain fluctuation over the coverage from −30° to 30° as a benchmark of the beam-forming effect. Here, the gain fluctuation is defined as the numerical difference between the maximum and minimum values of the gain, within the ±30° coverage. Table 2 shows the change in gain fluctuation while the included angle is increasing from 20° to 40°. For ETC antennas, small gain fluctuation means higher communication stability and lower communication interruption. Hence, the best value for $\theta_{1}$ and $\theta_{2}$ is optimized to be 33°, according to Table 2, and the corresponding gain fluctuation is only 0.74 dB, which is within the required angular coverage from −30° to 30°. As reported by (7), the optimal distribution of excitations can be calculated and is listed in Table 3. Obviously, it can be observed that the amplitude and phase of the 16 elements are completely different. The eight elements in the middle, numbered 5 to 12, contribute the most to the overall beam shaping, with their combined power exceeding 95%. Although the other eight antenna elements share 5% power, they are essential for the flat-top-shaping effect.

### 3.3. Feeding Network Design

**Figure 6:**
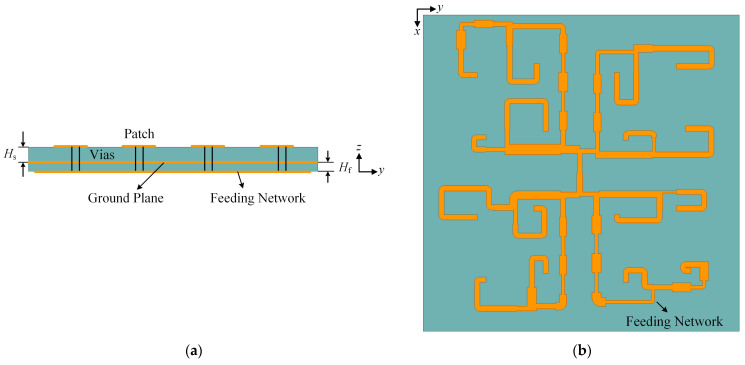
Different views of the proposed design. (**a**) Side view; (**b**) Back view.

In order to feed the 16 antenna elements with the optimized distribution of excitations, a well-designed feeding network, based on the microstrip transmission line theory, is indispensable. Considering that the array element is a center-fed microstrip antenna, a double-layered structure is used in the feeding network design. As is shown in Figure 6a, the upper layer is the antenna array with a thickness *H*_s_ of 2 mm, and the lower layer is the feeding network printed on the FR4 substrate with a thickness *H*_f_ of 1 mm. Thus, the ground plane in the middle is shared by the microstrip antenna and the feeding network plotted in Figure 6b.

**Figure 7:**
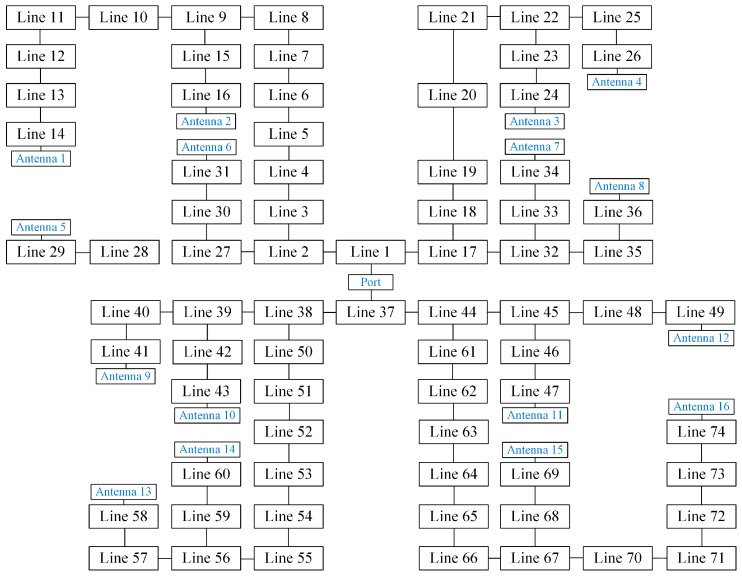
Schematic diagram of the proposed feeding network.

The proposed microstrip feeding network consists of several power dividers, phase delay lines, and quarter-wavelength impedance converters. All these parts can be equivalent to the microstrip lines, with determined widths and lengths. As illustrated in Figure 7, the proposed 1-to-16 feeding network is the combination of 74 microstrip lines whose widths and lengths are shown in Table 4. To avoid the loss caused by lines with excessive impedance differences, more quarter-wavelength impedance converters are applied to deal with the situation of branches with high power allocation ratios. As a result, the line width of the proposed feeding network is controlled to be within the range of 0.8 mm to 3.3 mm.

To ensure the shaping effect, the differences of the amplitudes and phases between the feeding network and the optimized excitations are strictly controlled to be smaller than 0.01 V and 1°. Table 5 shows the excitations accomplished by the proposed feeding network, which are consistent with the optimized values of excitations in Table 3.

## 4. Results and Discussion

**Figure 8:**
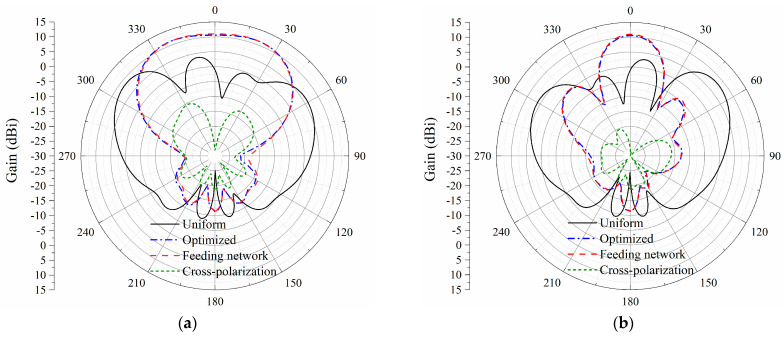
Radiation patterns of the 16-element antenna array under different excitations. (**a**) *xz*-plane; (**b**) *yz*-plane.

While the distribution of excitations is determined and accomplished by a well-designed feeding network, the simulated far-field performances are obtained and shown in Figure 8. In can be seen from Figure 8 that the radiation patterns both on the *xy*-plane and the *yz*-plane are unorganized under the condition of uniform excitations.

**Figure 9:**
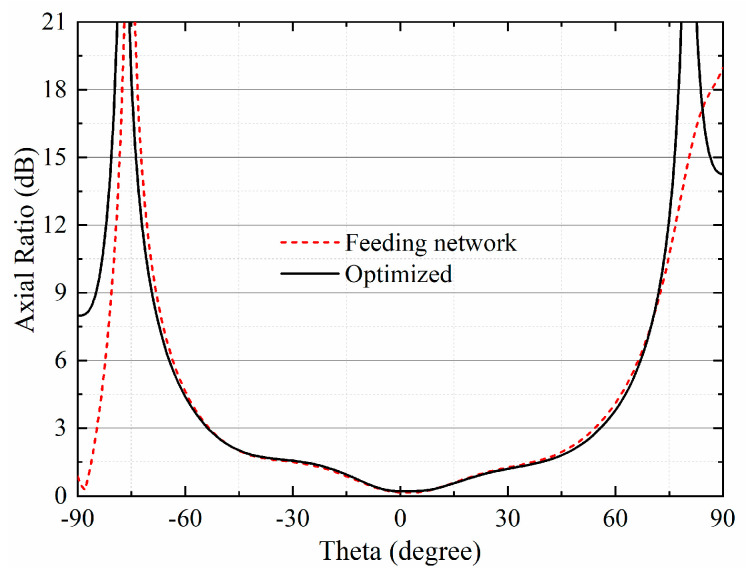
Axial ratio of the 16-element antenna array in *xy*-plane.

**Figure 10:**
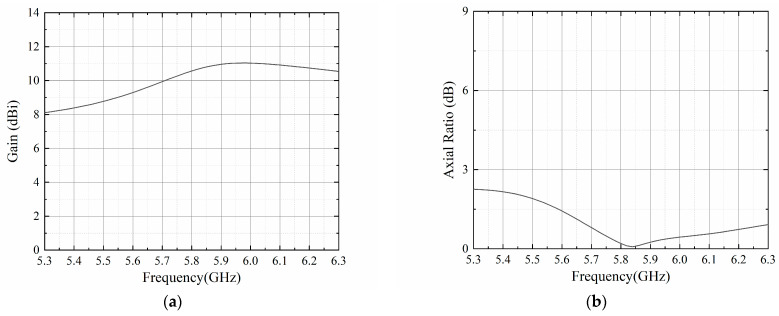
(**a**) Gain and (**b**) axial ratio variations with the change in frequency.

The 16-element antenna array with a feeding network provides a flat-top sector beam on the *xy*-plane. Within the required angular coverage from −30° to 30°, a maximum gain of 11.3 dBi is achieved, while the gain fluctuation is lower than 0.7 dB. Furthermore, the radiated power is concentrated in the main beam, leading to no sidelobes on the upper half plane. As for the *yz*-plane, the simulated half-power beamwidth (HPBW) is 24.4°. The sidelobe is directed to −44°, with the side lobe level below −11.4 dB. As is shown in Figure 9, the simulated AR remained below 3 dB from −53° to 55°. Within the required 60° range, the AR is only lower than 1.5 dB, indicating a good circularly-polarized performance. Figure 10 shows the gain and AR variation with the change in frequency within a 1 GHz range around the center frequency. The gain is maintained over 10 dBi from 5.7 GHz to 6.3 GHz, with a peak gain of 11 dBi at 5.9 GHz. The AR remains below 3 dB, while the frequency changes from 5.3 GHz to 6.3 GHz.

**Figure 11:**
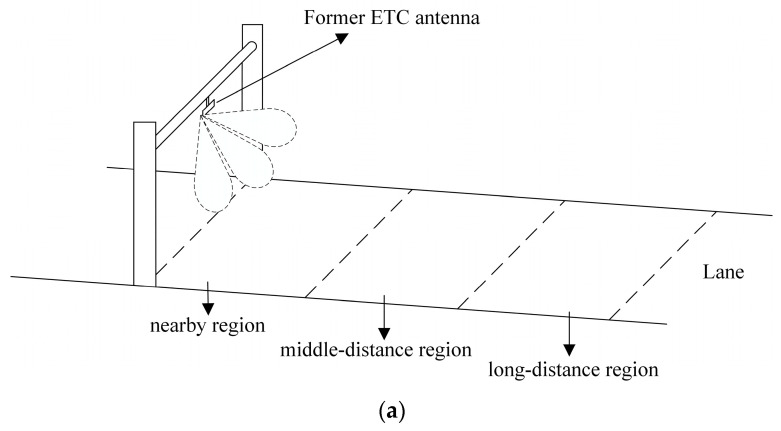

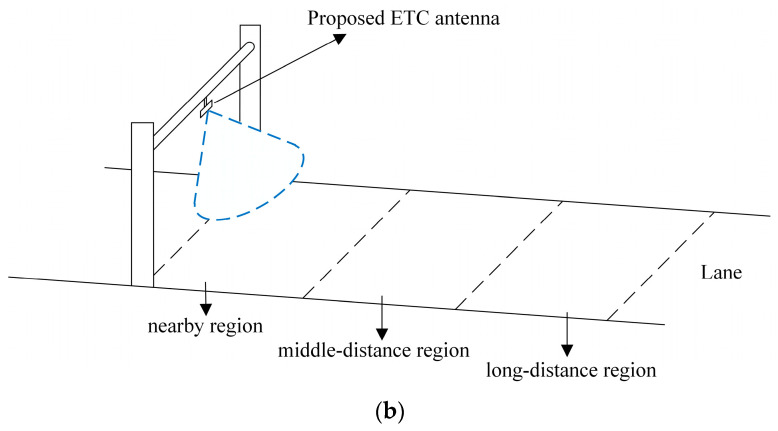
Schematic diagram of the ETC system with (**a**) former ETC antenna and (**b**) the proposed ETC antenna.

In the current commercial ETC system, the required angular coverage for wireless communication ranges from −30° to 30° in the vertical plane. As is shown in Figure 11, the former approach to achieve the whole 60° coverage is to use a beam-switching method depending on the vehicle’s position in the near-lane region, middle-distance region, and long-distance region. However, two drawbacks may occur during the beam-switching process. One problem is the short communication interruption at the boundary of adjacent regions, caused by the short beam switching time. The other, is the unstable signal, because the antenna gain continuously decreases outward from the single-beam direction. To solve the above-mentioned issues, the proposed design suggests generating a flat-top sector beam, covering the −30° to 30° range, with a gain fluctuation of only 0.7 dB, leading to much more stable and continuous communication. Furthermore, it should be noted here that the angular coverage can be flexibly adjusted by changing the number, as well as the positions of the test antennas, which is suitable for potential applications with different requirements. The test receiving antennas here only serve the purpose of shaping the antenna array to be designed, and do not require real manufacture, which is also to the benefit of the presented method.

Performance comparisons of the proposed design and other publications have been made in Table 6 regarding the frequency, gain fluctuation among the required coverage, minimum AR, 3-dB AR beam coverage, sidelobe level, and antenna size. The proposed design has a minimum AR and wider 3-dB AR beam coverage, resulting in better circular polarization performance. Furthermore, the gain fluctuation of the proposed 16-element circularly polarized antenna array among the required angular coverage from −30° to 30° is only less than 0.7 dB, which suggests a higher communication stability.

## 5. Conclusions

In this paper, an optimization theory called WCMMPTE is proposed for the design of beam-shaping antenna arrays. To verify the validity of WCMMPTE, a 4 × 4 circularly-polarized planar microstrip patch antenna array, operating at 5.8 GHz (ISM band), is designed. By setting the equality constraints on the received power of the test receiving antennas, the beam shaping problem can be viewed as a QCQP problem. Through applying the Lagrange multiplier method, the solution is in an analytical form, which reveals the optimal distribution of excitations for the antenna array. The simulation results show that a flat-top sector beam is generated by feeding the antenna elements with a well-designed feeding network. Within the required angular coverage from −30° to 30°, a maximum gain of 11.3 dBi is achieved, while the gain fluctuation is lower than 0.7 dB, and the AR is lower than 1.5 dB. In addition, there is no sidelobe in the *xz*-plane, and the side lobe level is below −11.4 dB in the *yz*-plane, which avoids the misreading of the surrounding area. In summary, the proposed design based on WCMMPTE can provide a stable signal and uninterrupted communication, which is suitable for the current electronic toll collection system.

## Figures and Tables

**Table 1:** Parameters for the microstrip patch antenna.

Parameter	Value
*W* (mm)	11.3
*L* (mm)	11.6
*s* (mm)	1.88
*d* (mm)	2.57

**Table 2:** Gain fluctuation under different selections of included angles.

$\mathrm{Value}\;\mathrm{for}\;\theta_{1}$$\;\mathrm{and}\;\theta_{2}$ (Degree)	Gain Fluctuation (dB)
20	2.47
27	0.86
30	1.31
33	0.74
36	1.08
40	2.27

**Table 3:** Optimized distribution of excitations.

Antenna No.	Optimized Excitation
1	0.04 V < 51.8°
2	0.09 V < 154.1°
3	0.06 V < −89.6°
4	0.10 V < 147.2°
5	0.37 V < 148.2°
6	0.28 V < −114.5°
7	0.35 V < 139.4°
8	0.35 V < −113.0°
9	0.33 V < 68.4°
10	0.39 V < 31.0°
11	0.32 V < 48.9°
12	0.37 V < −26.1°
13	0.06 V < −32.0°
14	0.07 V < 48.4°
15	0.07 V < −13.2°
16	0.04 V < 85.6°

**Table 4:** Widths and lengths for the microstrip lines.

Line No.	Width (mm)	Length (mm)	Line No.	Width (mm)	Length (mm)	Line No.	Width (mm)	Length (mm)
1	1.75	7.04	26	1.89	40.61	51	2.71	6.89
2	2.93	6.86	27	3.77	20.30	52	1.37	7.13
3	1.66	7.06	28	1.62	7.07	53	2.71	6.89
4	2.71	6.89	29	1.89	13.02	54	1.17	7.18
5	1.01	7.23	30	1.62	7.07	55	2.71	6.89
6	3.30	6.81	31	1.89	35.50	56	1.89	7.02
7	1.37	7.13	32	2.51	20.74	57	1.62	7.07
8	2.71	6.89	33	0.92	7.25	58	1.89	28.15
9	1.74	21.14	34	1.89	14.24	59	3.36	6.81
10	1.57	7.08	35	1.95	7.01	60	1.89	21.35
11	2.71	6.89	36	1.89	33.29	61	2.09	6.98
12	1.37	7.13	37	2.04	6.99	62	3.30	6.81
13	3.52	6.79	38	2.10	6.98	63	1.17	7.18
14	1.89	15.36	39	2.99	20.50	64	3.30	6.81
15	2.41	6.93	40	2.10	6.98	65	1.17	7.18
16	1.89	29.72	41	1.89	49.76	66	3.30	6.81
17	1.66	7.06	42	1.39	7.12	67	1.17	21.54
18	1.37	7.13	43	1.89	28.13	68	2.00	7.00
19	2.71	6.89	44	1.46	7.11	69	1.89	13.27
20	1.37	7.13	45	2.39	20.80	70	1.82	7.03
21	2.71	6.89	46	2.17	6.97	71	3.30	6.81
22	1.37	21.38	47	1.89	21.39	72	1.17	7.18
23	1.68	7.06	48	0.80	7.29	73	3.03	6.85
24	1.89	29.24	49	1.89	25.94	74	1.89	11.22
25	2.76	6.88	50	1.77	7.04			

**Table 5:** Excitations accomplished by the feeding network.

Antenna No.	Optimized Excitation
1	0.03 V < 51.8°
2	0.08 V < 154.1°
3	0.05 V < −89.6°
4	0.9 V < 147.2°
5	0.36 V < 148.2°
6	0.27 V < −114.1°
7	0.34 V < 139.4°
8	0.35 V < −113.0°
9	0.34 V < 68.9°
10	0.40 V < 32.9°
11	0.33 V < 49.0°
12	0.39 V < −26.9°
13	0.06 V < −32.2°
14	0.06 V < 47.4°
15	0.06 V < −12.9°
16	0.03 V < 86.5°

**Table 6:** Performance comparison of the proposed design and other publications.

Ref.	Frequency (GHz)	Gain Fluctuation (dB)	Minimum AR (dB)	3-dB AR Beam Coverage	Sidelobe Level (dB)	Antenna Size
[7]	5.8	12.9	0.3	(−23°, 23°)	<−30	120 mm × 125.2 mm × 2 mm (2.22 λ × 2.42 λ × 0.04 λ)
[8]	0.92	13	<2	Not given	<−22	590 mm × 590 mm × 1.524 mm (1.81 λ × 1.81 λ × 0.005 λ)
[9]	3.8	>1	1.8	(−18°, 40°)	<−8	138 mm × 138 mm × 6.15 mm (1.75 λ × 1.75 λ × 0.08 λ)
This work	5.8	<0.7	0.202	(−54°, 56°)	<−11.4	114.7 mm × 115 mm × 3 mm (2.22 λ × 2.22 λ × 0.06 λ)

## Data Availability

Data are contained within the article.
